# Targeting GABA signaling in the tumor microenvironment: implications for immune cell regulation and immunotherapy resistance

**DOI:** 10.3389/fimmu.2025.1645718

**Published:** 2025-08-05

**Authors:** Yuanqing Zhao, Jin Xu, Ke Yang, Li Bao

**Affiliations:** ^1^ Department of Laboratory Medicine, Clinical Laboratory Medicine Research Center, West China Hospital, Sichuan University, Sichuan Clinical Research Center for LaboratoryMedicine, Chengdu, Sichuan, China; ^2^ Department of Laboratory Medicine, Chengdu Shangjin Nanfu Hospital, Chengdu, Sichuan, China; ^3^ Medicine Research Center, West China Hospital, Sichuan University, Sichuan Clinical Research Center for Laboratory Medicine, Chengdu, Sichuan, China; ^4^ Department of Radiology, Key Laboratory of Birth Defects and Related Diseases of Women and Children of Ministry of Education, West China Second University Hospital, Sichuan University, Chengdu, China

**Keywords:** GABA signaling, tumor immunotherapy, tumor microenvironment, immune cell reg ulation, cancers

## Abstract

As an important inhibitory neurotransmitter, γ-aminobutyric acid (GABA) not only plays a key role in the central nervous system, but also has attracted wide attention in the tumor immune microenvironment in recent years. Studies have shown that tumor cells can synthesize GABA and use it to remodel the tumor microenvironment, thereby promoting the occurrence, development and metastasis of tumors. Although previous studies have revealed the important role of GABA in tumor immune escape, there are still many unknown areas of its mechanism, especially the heterogeneous manifestations in different tumor types and tissue environments. This review summarizes the immunomodulatory mechanisms of GABA in tumor-associated macrophages, CD8^+^ T cells and dendritic cells in the tumor immune microenvironment, and discusses its potential role in tumor immune escape and immunotherapy resistance, providing new ideas for the development of immunotherapeutic drugs targeting GABA receptors.

## 1 Introduction

Cancer remains a major global public health challenge, with incidence and mortality rates continuing to rise. According to the “Cancer Statistics, 2024” report, over 19 million new cancer cases were diagnosed worldwide in 2023, with certain tumor types such as breast cancer and lung cancer maintaining high incidence rates and showing significant variability in five-year survival rates among patients (1). Reviewing the history of cancer treatments, based on the NIH’s “Cancer Treatments: Past, Present, and Future” (2024), therapeutic approaches have evolved from traditional surgery and radiotherapy to chemotherapy, followed by targeted therapies and immunotherapies, greatly improving patient outcomes (2, 3). However, current treatments still face challenges posed by tumor heterogeneity and therapeutic resistance. Regarding cancer treatment strategies, recent research in “Different Strategies for Cancer Treatment: Targeting Cancer Cells or Their Neighbors?” (2025) highlights two primary approaches: direct targeting of tumor cells and modulation of the tumor microenvironment (4). The former achieves rapid tumor cell killing by targeting tumor-specific molecules but is prone to resistance development; the latter improves immune infiltration by regulating immune cells and stromal components, with complex mechanisms but considerable potential. Integrative treatment strategies that combine the advantages of both approaches are considered the future direction in oncology.

γ-Aminobutyric acid (GABA), a principal inhibitory neurotransmitter in the central nervous system, plays a critical role in modulating neuronal excitability, exerting calming and sedative effects (5). Traditionally recognized for its neurological functions, GABA has recently garnered attention in oncology due to its aberrant upregulation in a variety of solid tumors, where elevated GABA levels have been correlated with poor clinical outcomes (6). Emerging evidence indicates that tumor cells not only possess the capacity to synthesize GABA but also exploit it to modulate the tumor microenvironment, thereby promoting tumor progression and metastasis (7).

Within the landscape of tumor immune evasion, cancer cells deploy multifaceted mechanisms to escape host immune surveillance, ultimately undermining the efficacy of immunotherapy (8). A well-characterized strategy involves the upregulation of immune checkpoint molecules such as programmed death-ligand 1, which inhibits T cell activation and effector function (9). Beyond checkpoint pathways, GABA has been shown to reprogram tumor-associated macrophages toward an immunosuppressive M2 phenotype, thereby fostering an immune-permissive tumor microenvironment (10). Moreover, GABA signaling through GABA_A_ receptors can directly impair the cytotoxic activity of CD8^+^ T cells and reduce their production of interferon-gamma (IFN-γ). Simultaneously, GABA facilitates the infiltration and activity of regulatory T cells (Tregs), contributing to a multilayered immune evasion network (11).

Therefore, it is of great significance to further study the immunomodulatory role of GABA in the tumor microenvironment. A deeper understanding of how GABA mediates immune escape and resistance to immunotherapy may not only shed light on the biological underpinnings of cancer progression but also pave the way for innovative therapeutic strategies. This review aims to examine the emerging role of GABA in tumor immune evasion and immunotherapy resistance, and to explore its implications for the future of cancer immunotherapy.

## 2 Mechanisms and functions of GABA signaling

**Figure 1:**
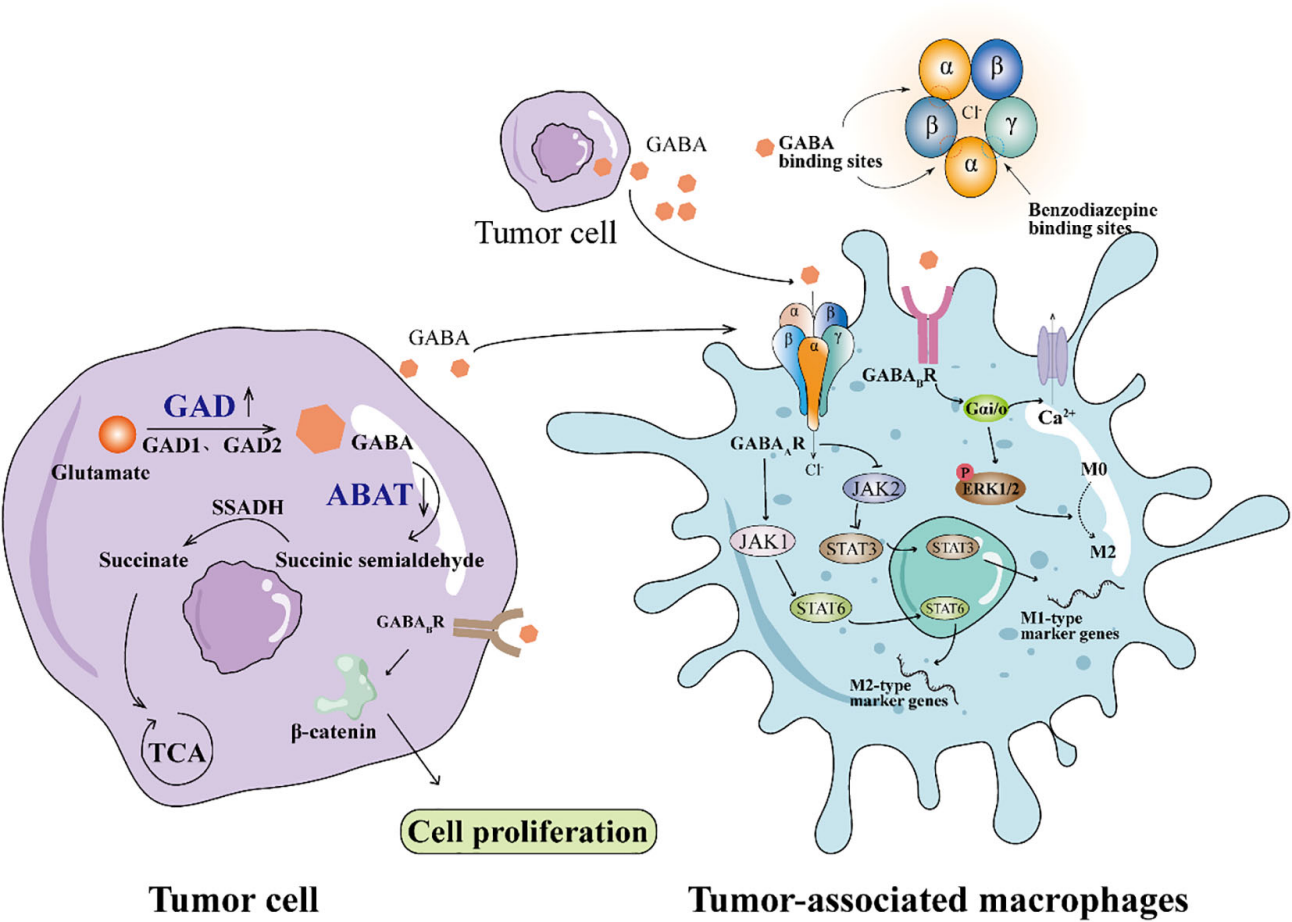
GABA signaling pathway in tumor microenvironment stimulates the polarization of macrophages to M2 type. Downregulation of GAD expression and downregulation of ABAT expression in tumor cells leads to GABA accumulation. GABA_A_R on the surface of TAMs binds GABA from tumor cells to activate JAK1/STAT6 signaling and promote M2 polarization. GABA couples GABA_B_R to Gαi, activates ERK1/2 signaling pathway, and promotes M2 polarization.

Recent studies have demonstrated that the role of GABA signaling within the tumor immune microenvironment shows significant heterogeneity across different cancer types and tissue contexts. For example, in glioblastoma, activation of the GABAB receptor on granulocytic myeloid-derived suppressor cells (gMDSCs) enhances L-arginine metabolism and NOS2 expression, promoting tumor growth; conversely, inhibition of GABABR prolongs survival in mouse models (12). In breast cancer, GABAergic signaling modulates tumor progression through distinct mechanisms: activation of the GABAA receptor δ subunit (GABRD) enhances GPT2-mediated metabolic reprogramming, promoting metastasis, while upregulation of the β3 subunit fosters clonal expansion and cell migration in triple-negative breast cancer (13). In pancreatic ductal adenocarcinoma (PDAC), GABA has been shown to promote proliferation via the GABAA receptor π subunit; paradoxically, some studies suggest it may suppress migration, indicating a complex bidirectional role. Moreover, in hepatocellular carcinoma (HCC), GABAergic signaling through distinct receptor subunits (such as α3, β3, π, θ) may exert context-dependent effects, with certain receptor profiles linked to tumor suppression (13). Recent studies have revealed significant differences in the expression of various GABA receptor subunits within immune cells. For example, the expression levels of the α3 and β2 subunits differ between T cells and tumor-associated macrophages (TAMs), which may lead to distinct responses to GABA signaling. Specifically, the α3 subunit may be involved in regulating immune cell activation and cytokine secretion, whereas the β2 subunit may influence cell polarization and immunosuppressive functions (10, 14). Selective modulation of different subunits not only helps to uncover the complex roles of GABA signaling in immune regulation but also provides a theoretical basis for the development of highly selective targeted drugs, thereby enabling precise immunotherapy strategies that improve efficacy while minimizing side effects. Collectively, these findings emphasize that the immunomodulatory effects of GABA are highly context-dependent and are shaped by tumor-intrinsic characteristics, tissue-specific microenvironments, and the composition of immune cell populations. GABA synthesis primarily depends on glutamate decarboxylase (GAD), which converts glutamate into GABA. GAD exists in two isoforms, GAD65(GAD2) and GAD67(GAD1), which play roles at the nerve terminals and in areas such as the cerebral cortex, respectively (15, 16). After synthesis, GABA undergoes metabolism via GABA transaminase (ABAT), which converts it into succinic semialdehyde, which is further transformed into succinate by succinic semialdehyde dehydrogenase (SSADH), entering the tricarboxylic acid(TCA) cycle for energy metabolism(**Figure 1**). Previous studies have observed GAD1 upregulation and ABAT downregulation in tumor cells (6). Additionally, GABA is taken up by glial cells and neurons through GABA transporters (GAT) and is either converted back into glutamate or used for the synthesis of new GABA (17). The balance between GABA synthesis and metabolism is crucial for the proper function of the nervous system, and its dysregulation is closely associated with various neurological disorders, such as epilepsy and anxiety (18, 19).

The biological effects of GABA are primarily mediated through its receptors, including GABA_A_, GABA_B_, and GABA_C_ receptors (20–22). GABA_A_ receptors are ion channel receptors, and when GABA binds to them, Cl^-^ flow into the cell, causing hyperpolarization of the neuron and inhibiting neural activity, thus mediating rapid inhibitory synaptic transmission (23). GABA_A_ receptors are drug targets, and benzodiazepines enhance their effects, providing sedative and anti-anxiety properties (24, 25). In contrast, GABA_B_ receptors are G-protein coupled receptors, which regulate Ca^2+^ and K^+^ channels to produce slower inhibitory effects, involved in long-term neural regulation, and play a broad role in learning, memory, and pain control (26, 27). GABA_C_ receptors, primarily located in the retina (28), are ion channel receptors responsible for the rapid inhibitory transmission of visual information. These three receptor types each play a unique role in the nervous system, collectively maintaining the balance of neural function.

## 3 Role of GABA in tumor microenvironment

Tumor immune evasion refers to the ability of tumor cells to evade recognition and clearance by the host immune system, allowing the tumor to survive and develop under immune surveillance (29, 30). The primary function of the immune system is to maintain the health of the body by recognizing and attacking foreign substances, mutated cells, and tumor cells. However, tumor cells avoid this clearance by altering their surface characteristics or by modulating the immune environment. Tumor immune evasion is one of the major causes of cancer resistance and the progression of malignant tumors (31). Through immune evasion, tumor cells not only avoid detection by the immune system but may also accelerate tumor growth, metastasis, and the development of resistance to treatment. Studies have shown that GABA stimulates tumor cell proliferation by activating GABA_B_ receptors (32). This process involves the inhibition of the GSK-3 signaling pathway, leading to the activation of β-catenin, which subsequently inhibit tumor cell proliferative capacity and impairs CD8^+^ T cell infiltration into tumors (6, 33). Mohita et al. (34) found that GABA promotes melanoma development by releasing a SNAR-dependent vesicle pathway. GABA signaling has an immunosuppressive effect in the tumor microenvironment, which makes GABA receptors a potential therapeutic target for cancer immunotherapy (35).

## 4 The influence of GABA on immune cells

### 4.1 The effect of GABA on TAM

Tumor-associated macrophages (TAMs) are important immune cells within the tumor microenvironment, primarily originating from circulating monocytes (36, 37). Tumors recruit these monocytes by secreting chemokines such as CCL2 and growth factors like CSF-1. These recruited monocytes differentiate into macrophages and become TAMs in response to local signals. The functional phenotype of TAMs is highly plastic and influenced by multiple signals within the tumor microenvironment. Pro-inflammatory stimuli such as IFN-γ and LPS drive TAMs toward an M1 phenotype, characterized by anti-tumor activity through the production of inflammatory cytokines and antigen presentation (38). In contrast, anti-inflammatory cytokines including IL-4 and IL-13 promote M2 polarization, which supports tumor progression by facilitating immune suppression, angiogenesis, and tissue remodeling. Increasing evidence suggests that GABA signaling can modulate TAM polarization. GABA binds to GABA receptors on macrophages, leading to downstream signaling changes that favor M2 polarization. Dong et al. (39) showed that macrophages activate the JAK1/STAT6 signaling pathway via GABA, which promotes the expression of Arg1, a gene related to M2. GABA aiso inhibited the NF-κB and JAK2/STAT3 signaling pathways, and decreased the expression of iNos related to M1 (**Figure 1**). This finding is consistent with previous research, and Zhang et al. (40) also confirmed that GABA can significantly reduce the nuclear localization of p65 in the NF-κB signaling pathway.

The changes in GABA_A_ receptor (GABA_A_R) expression may form the basis for macrophage polarization. During the differentiation of monocytes into the M0 macrophage phenotype, the expression of the GABA_A_R β2 subunit increases significantly, while the expression of the GABA_A_R α4 subunit shows no significant change. When M0 macrophages polarize to the M1 phenotype, the expression of the GABA_A_R α4 subunit and GAD1 decreases, while the β2 subunit expression returns to monocyte levels, and the expression of GAT2 increases significantly (41). These changes diminish the response of GABA_A_R to GABA, thereby relieving GABA’s anti-inflammatory suppression and supporting the pro-inflammatory function of M1 macrophages. GABA Transporters(GAT) belongs to the SLC6A family, which includes GAT1–4 and is responsible for transporting GABA from the synaptic cleft or extracellular environment to the cell to maintain GABA homeostasis.Xia et al. (42) GAT2 deficiency in macrophages can increase intracellular betaine content, leading to hypoxanthine and S-adenosylmethionine accumulation (SAM), and the intracellular betaine/SAM/hypoxanthine metabolic pathway affects the methylation of the transcription factor KID3. It inhibits the formation of NLRP3-ASC-Caspase-1 complex and increases the intracellular OXPHOS level, thereby inhibiting the production of IL-1β in M1 macrophages. Liu et al. (43) found that GABA receptor agonists can promote the polarization of macrophages towards the M2 phenotype. Sun et al. (44) found that GABA-related genes can be used to judge the prognosis of glioma. Dou et al. (45) found that GABA secreted by oral squamous cell carcinoma (OSCC) promotes macrophage polarization toward the M2 phenotype by activating the GABA_B_ R1 and its downstream ERK and Ca²^+^ signaling pathways, which promoted EMT of OSCC *in vivo*(**Figure 1**). These results suggest that GABA may play a role in tumor immune escape by regulating the polarization of macrophages in the tumor microenvironment.

### 4.2 The effect of GABA on T cell

**Figure 2:**
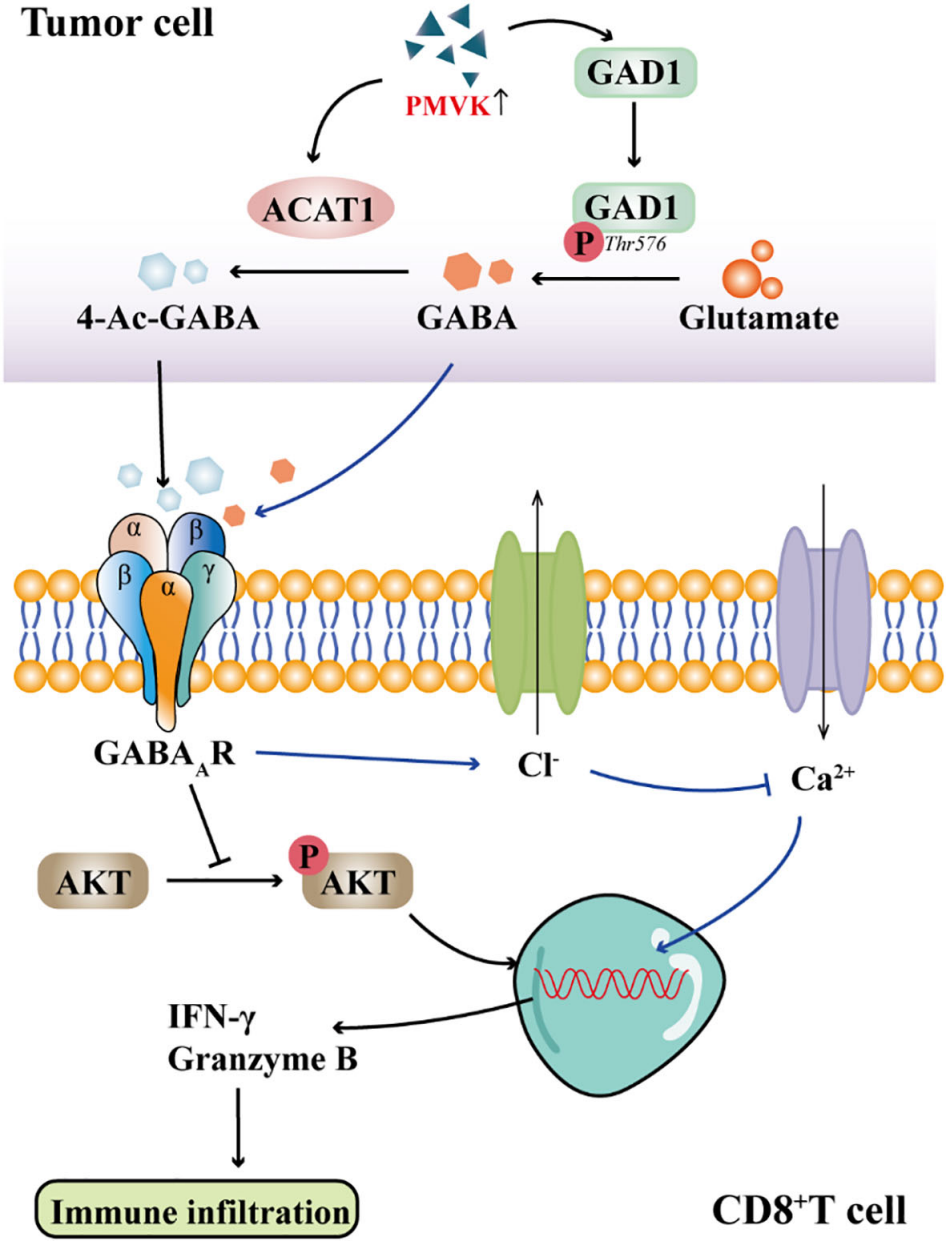
GABA signaling inhibits CD8+T cell immune infiltration. GABAAR on the membrane of CD8+T cells binds to 4-Ac-GABA from tumor cells, inhibits the phosphorylation of AKT and prevents the production of IFN-γ and Granzyme B, thereby affecting T cell activation and immune infiltration. Activation of GABA_A_R results in Cl^−^ efflux, inhibition of Ca^2+^ influx, and ultimately inhibition of T cell activation.

CD8^+^ T cells are a major subset of cytotoxic T lymphocytes, which express CD8 coreceptors and recognize antigens through MHC class I molecules. In the tumor microenvironment, CD8^+^ T cells are the core effector cells of anti-tumor immunity, and their activity is positively correlated with the prognosis of patients. Tharp et al. (46) found that in the fibrotic tumor microenvironment, TAMs initiate collagen biosynthesis through TGF-β signaling, creating a metabolic environment that depletes arginine and secretes proline and ornithine, thereby inhibiting CD8^+^ T cell antitumor responses. Previous studies have found that PMVK expression is increased in tumor tissues (47). Zhou et al. (11) observed in hepatocellular carcinoma cells that PMVK activity is negatively correlated with CD8^+^ T cell infiltration and immune evasion. Their study demonstrated that PMVK promotes the conversion of glutamate to GABA by phosphorylating threonine 576 at the C-terminus of GAD1, thereby increasing GABA synthesis. Furthermore, PMVK directly binds to and stabilizes ACAT1 protein, facilitating the acetylation of GABA to generate 4-Ac-GABA. 4-Ac-GABA binds to the α3 subunit of the GABA_A_R on the surface of CD8^+^ T cells, inhibiting AKT1 phosphorylation, which subsequently reduces the expression of CD8^+^ T cell activation markers such as IFN-γ and granzyme B, and decreases their infiltration into the tumor microenvironment (**Figure 2**). Immunofluorescence conducted by Sparrow et al. (48) confirmed the presence of GABA_A_R subunits on the surface of both mouse and human T cells. Mouse T cells predominantly express the α2, α3, α5, β2, β3, γ1, and δ subunits, whereas human T cells highly express the α1, α5, β1, π, ρ1, and ρ2 subunits, with the ρ2 subunit being enriched on the surface of human T cells. Activation of GABA_A_R by benzodiazepines or neurosteroids significantly inhibited the proliferation of both mouse and human T cells, and this effect was reversed by GABA_A_ receptor antagonists. These findings indicate that GABA_A_ receptors play a crucial role in regulating T cell function; their activation can suppress T cell proliferation and immune activity, thereby potentially contributing to the regulation of immune responses and tumor immune evasion.

The enhancement of Ca^2+^ signaling can accelerate the activation process of T cells, thereby improving their responsiveness to tumor cells. Zhang et al. (40) implanted sustained-release GABA particles into muMt^-/-^ mice and found that tumor growth in the mice was significantly increased. Further investigation revealed that GABA might regulate CD8^+^ T cell function by binding to the GABA_A_R on the surface of T cells, inhibiting Ca^2+^ influx.Consistent with these findings, Tian et al. (49) also discovered that GABA activates GABA_A_R on the surface of T cells, leading to the opening of Cl^-^ channels, Cl^-^ efflux, and membrane depolarization, which in turn inhibits Ca^2+^ influx (**Figure 2**). Ca^2+^ influx is a critical step for the activation of naïve T cells and the function of effector T cells. Activation of GABA receptors primarily causes T cells to arrest in the G_0_/G_1_ phase, preventing their entry into the proliferative cycle and thereby inhibiting clonal expansion. Although the expression of GABA_A_R subunits has been identified in T cells, the specific role of these subunits in regulating T cell function is not well defined. The effects of different subunits on T cell activity, polarization and immune escape may be different, and further studies are needed to clarify.

### 4.3 The effect of GABA on DCs

Dendritic cells (DCs) are key antigen-presenting cells that bridge innate and adaptive immunity. They capture and internalize foreign antigens, process them into peptide fragments, and present these peptides bound to major histocompatibility complex (MHC) molecules to T cell receptors (50). This process activates and directs T cells to mount a targeted immune response against specific pathogens or abnormal cells. Dendritic cells promote the recognition and elimination of tumor cells by activating CD8^+^ cytotoxic T cells and CD4^+^ helper T cells (51). In addition, Dcs regulate the immune microenvironment, influencing the development of immune tolerance and immune evasion. Tumor cells and their surrounding stroma secrete various immunosuppressive factors, such as TGF-β、IL-10 and VEGF, which inhibit the maturation and activation of dendritic cells, reducing their antigen-presenting capacity (52). In addition, metabolic products in the tumor microenvironment, such as lactate, can also suppress DCs function (53). Studies have shown that GABA receptors are not only present on T cells, B cells, and macrophages, but their expression profile also includes DCs (54). GABA is synthesized from glutamate catalyzed by GAD, which is expressed in DCs, indicating that DCs have the ability to produce GABA. At the same time, DCs express GATs on their surface, such as GAT1 and GAT3, enabling them to uptake extracellular GABA (55). Bekić et al. (56) found that in DCs, activation of the GABA_B_R induces a conformational change that couples with intracellular Gαi proteins, thereby activating G protein-coupled signaling pathways and inhibiting adenylate cyclase activity. This leads to a reduction in intracellular cAMP levels. The lowered cAMP levels promote the transition of DCs from an “immature” to a “mature” phenotype, increasing the expression of MHC II, CD86, and CD40, as well as the secretion of pro-inflammatory cytokines such as IL-1β, which in turn induces T cell differentiation toward the Th1 phenotype. This study reveals that monocyte-derived DCs may promote T cell proliferation and Th1 polarization through the GABA_B_R/cAMP signaling pathway. Similarly, Huang et al. (57) also demonstrated that inhibiting the expression of GABA_B_R in DCs can suppress IL-6 production and hinder the differentiation of DCs into Th17 cells. Recent studies have further confirmed the regulatory role of GABA on dendritic cells (DCs) within the tumor microenvironment (TME). For example, one study demonstrated that tumor cells synthesize GABA through the expression of GAD1 and activate the β-catenin signaling pathway via GABA_B receptors, thereby inhibiting the recruitment of CD103^+^ DCs and the infiltration of T cells into the tumor, ultimately promoting immune evasion and tumor progression (6). These findings indicate that GABA not only exerts effects in *in vitro* monocyte-derived DC cultures but also modulates anti-tumor immune responses by affecting DC function within the tumor microenvironment. In summary, while the role of GABA in regulating T cells and macrophages has been relatively well studied, its effects on dendritic cells—especially within the tumor microenvironment—remain underexplored. Current evidence suggests that GABAergic signaling influences DC maturation and cytokine production, thereby shaping downstream T cell polarization. However, the specific consequences of this modulation in cancer settings, including its contribution to immune evasion or resistance to immunotherapy, warrant further investigation. In addition, Current research on the regulation of dendritic cells (DCs) by GABA is limited and yields somewhat contradictory results. On one hand, some studies suggest that GABA can promote DC maturation and antigen-presenting functions, thereby enhancing immune activation. On the other hand, other studies indicate that GABA may inhibit DC activity, reducing their capacity to stimulate T cells. These conflicting findings may be due to influences from different tumor types and microenvironmental factors, such as local cytokine levels, metabolic states, and interactions with other immune cells. Additionally, variations in experimental models and methodologies may also contribute to inconsistent observations. In summary, a deeper investigation into the specific effects of GABA on DCs within diverse tumor microenvironments is crucial for understanding its immunoregulatory roles and for developing related immunotherapeutic strategies.

## 5 GABA signaling pathway plays a key role in the mechanism of tumor immunosuppression and drug resistance

Studies have shown that GABA can upregulate the expression of PD-L1 on the surface of tumor cells by activating the STAT3 signaling pathway, thereby suppressing anti-tumor immune responses (58). PD-L1 is an immune checkpoint protein expressed on tumor cells that binds to the PD-1 receptor on T cells, inhibiting their activity and promoting tumor immune evasion. As a key immune checkpoint molecule, PD-L1 interaction with PD-1 on T cells suppresses T cell function, allowing tumor cells to escape immune surveillance (59, 60). STAT3 is a well-established oncogenic signaling pathway in various types of cancers, and its sustained activation is closely associated with tumor proliferation, metastasis, and immune evasion. One study demonstrated that GABA and its derivative baclofen can downregulate the mRNA and protein levels of the E3 ubiquitin ligase STUB1, thereby enhancing the stability of PD-L1 and ultimately increasing its expression (61). This mechanism indicates that GABA not only functions within the nervous system but may also influence tumor immune evasion in the tumor microenvironment by regulating the expression of immune checkpoint proteins. Moreover, the positive allosteric modulator of the GABA_A_R, QH-II-066, enhances GABA receptor function and can synergize with PD-L1 inhibitors to improve anti-tumor efficacy. In a mouse tumor model where PD-1 blockade therapy was ineffective, Huang et al. (6) found that the use of a GAD1 inhibitor alone, or in combination with an anti-PD-1 antibody, significantly reduced tumor volume. Switchenko et al. (62) found that benzodiazepines bind to the αγ subunit interface of the GABA_A_R, enhancing the binding efficiency of GABA to its receptor and significantly increasing the chloride ion permeability of the melanoma cell membrane, thereby promoting chloride influx into the cells. Following benzodiazepine treatment, melanoma cells exhibited mitochondrial membrane depolarization, leading to apoptosis, a process associated with the p53 signaling pathway and cytokine expression. Regarding cancer stem cells, the π subtype of the GABA receptor (GABRP) is highly expressed on the membrane surface of triple-negative breast cancer stem cells. Li et al. (63) found that GABRP maintains the membrane abundance of EGFR by inhibiting its lysosomal degradation. The sustained activation of EGFR promotes the phosphorylation of ERK (p-ERK), thereby enhancing the self-renewal and proliferation of cancer stem cells. More importantly, although conventional chemotherapeutic agents such as paclitaxel and doxorubicin can induce the enrichment of cancer stem cells, knockdown of GABRP reverses this effect, suggesting that GABRP is one of the key mediators of chemotherapy resistance. This study indicates that targeting GABRP may be a promising strategy to overcome immune resistance. In addition, GABA signaling is also closely associated with the recruitment of tumor-associated macrophages. In pancreatic ductal adenocarcinoma, Jiang et al. (64) found that although GABRP does not exert its oncogenic effects through the conventional GABA/Cl^-^ signaling pathway, it promotes the infiltration of immunosuppressive macrophages by interacting with KCNN4 and activating the Ca²^+^/NF-κB/CXCL5-CCL20 axis. This macrophage infiltration not only contributes to the formation of an immunosuppressive microenvironment but also impairs T cell function and cooperates with other mechanisms to induce resistance to immunotherapy. Tumor immune evasion and immunotherapy resistance are closely related but not entirely equivalent concepts. Tumor immune evasion primarily refers to the processes by which cancer cells avoid recognition and elimination by the immune system through mechanisms such as downregulating antigen expression, secreting immunosuppressive factors, and recruiting suppressive immune cells. In contrast, immunotherapy resistance specifically describes the phenomenon where tumors show no response or reduced efficacy following treatments like immune checkpoint inhibitors (e.g., anti-PD-1/PD-L1 antibodies). Recent studies have revealed that GABA signaling is involved not only in classical immune evasion but also in mediating immunotherapy resistance by regulating immune checkpoint molecule expression, promoting the accumulation of immunosuppressive cells (such as regulatory T cells and tumor-associated macrophages), and suppressing the activity of effector CD8^+^ T cells (11, 40, 65). For example, GABA, through activation of GABAB receptors and downstream pathways, enhances the immunosuppressive function of myeloid-derived suppressor cells, reduces infiltration and activity of anti-tumor T cells, thereby diminishing the efficacy of immunotherapy (66). Furthermore, currently, no GAD1-specific small-molecule inhibitors have entered cancer clinical trials. However, a preclinical study has shown that 3-mercaptopropionic acid (3−MPA), as a GAD1 inhibitor, can exert antitumor effects in osteosarcoma models by modulating the Wnt/β−catenin signaling pathway, suggesting that targeting GABA synthesis or metabolic pathways may hold therapeutic potential (67). Overall, these insights highlight the potential of targeting GABA signaling pathways as a novel strategy to overcome immunotherapy resistance.

## 6 Clinical translation and future perspectives

Despite the growing body of evidence implicating GABAergic signaling in tumor progression and immune modulation, the clinical translation of GABA-targeted therapies remains challenging. Most current findings are derived from *in vitro* studies or murine models, and several key barriers must be addressed before clinical application can be realized. First, off-target effects are a significant concern, especially given the widespread expression of GABA receptors and enzymes in both tumor and non-tumor tissues (68). Second, receptor-subtype selectivity remains poorly defined in the context of the tumor microenvironment (TME), where multiple GABA receptor subtypes may exhibit divergent roles across cell types. Third, the blood–tumor barrier (BTB) poses a major pharmacological obstacle, limiting the bioavailability of systemically administered agents to solid tumors. Finally, the dose-dependent neurotoxicity of GABAergic modulators, particularly in the central nervous system, necessitates careful safety evaluation. To move toward translational application, it is critical to integrate GABA-targeted strategies into the broader landscape of cancer immunotherapy. Recent studies have demonstrated how soluble PD-L1, cytokine profiles, and lymphocyte subsets can serve as circulating biomarkers to predict response to immune checkpoint inhibitors, particularly in melanoma (69). Moreover, next-generation CAR T-cell therapies are being engineered to resist hostile metabolic environments, such as hypoxia and high lactate, by enhancing mitochondrial resilience—thereby improving efficacy in solid tumors (70). In gastric cancer, evolving insights into the PD-1/PD-L1 axis have highlighted the need for combinatorial checkpoint blockade and personalized immunotherapy based on TME profiling (71). Additionally, interrupting extracellular vesicle-mediated communication between tumor cells and tumor-associated macrophages (TAMs) offers a novel approach to reprogramming the immunosuppressive TME (72). Combination strategies involving GABA-targeted therapies are an exciting and evolving frontier in cancer immunotherapy. Beyond PD-1/PD-L1 blockade, future research may explore the integration of GABA modulators with other immunotherapeutic approaches such as CTLA-4 inhibitors, CAR-T cells, and oncolytic viruses. These combinations may synergistically reshape the tumor immune microenvironment while mitigating adverse effects. Additionally, co-administration with metabolic reprogramming agents, cytokine inhibitors, or neuroprotective compounds may further enhance therapeutic efficacy and safety. Furthermore, it is important to acknowledge the ongoing role of traditional medicine, particularly in regions where herbal and mineral-based therapies remain widely used. Several recent studies have investigated both the mechanisms and clinical impact of traditional formulations, including the potential involvement of ion channels and metabolic pathways in the anticancer activity of so-called “toxic medicines” used in Traditional Chinese Medicine (TCM) (73, 74). Clinical reports have also demonstrated meaningful effects of traditional medicine on cancer outcomes (75, 76). Notably, biomarker-guided integration of traditional and modern therapies is emerging as a translationally viable direction. For instance, a recent study demonstrated that traditional medicine could counteract Trichostatin A–induced esophageal cancer progression when combined with biomarker-based strategies (77). These findings highlight the potential of holistic and multi-modal treatment paradigms in the context of GABA-targeted cancer immunotherapy. Taken together, the future of GABA-targeted cancer immunotherapy will likely depend on synergizing metabolic modulation with immunologic precision, as well as overcoming structural and pharmacodynamic barriers through advanced drug delivery and rational combination strategies.

## 7 Conclusion

In this review, we summarized the mechanisms of GABA synthesis and metabolism, as well as its immunosuppressive effects within the tumor microenvironment. We focused on how GABA influences tumor immune evasion and immunotherapy resistance by modulating key immune cells, including T cells, TAMs, and DCs. Although numerous studies have preliminarily revealed the pivotal role of GABA signaling in reshaping the tumor immune microenvironment and promoting immune evasion, its immunoregulatory mechanisms remain largely unclear. GABA suppresses the anti-tumor activity of various immune cells, including T cells and macrophages, but the specific receptor subtypes, signaling pathways, and functional differentiation involved remain to be fully elucidated. Moreover, the GABA pathway exhibits significant heterogeneity across different tumor types and tissue contexts. In most peripheral solid tumors, elevated expression of GAD1 and downregulation of GABA-degrading enzyme ABAT in tumor cells lead to the accumulation of GABA in the tumor microenvironment, thereby promoting tumor growth and immune evasion (78). In contrast, in brain metastases, upregulation of ABAT is frequently observed, facilitating the catabolism of neuron-derived GABA for tumor energy metabolism, suggesting that the function of GABA signaling varies dramatically depending on the tumor context. These contrasting roles likely arise from context-specific factors such as differences in GABA receptor subtype expression, microenvironmental signals (e.g., cytokines, cell types, neuronal innervation), and regional metabolic constraints (78). Understanding these determinants will be crucial for developing tissue-selective GABA-targeted strategies.

Emerging technologies such as single-cell RNA sequencing and spatial transcriptomics hold great promise in this regard, enabling high-resolution dissection of GABA signaling networks across diverse cellular subsets and anatomical regions (79, 80). For example, single-cell studies have identified novel exhausted CD8^+^ T-cell markers in breast cancer, revealed drug-tolerant persister cell vulnerabilities in colorectal cancer (81), and mapped glioma cell motility modulated by voltage-gated sodium channel β3 subunits (82). These insights underscore the power of single-cell approaches to uncover cellular heterogeneity and rare functional states. Compared to traditional bulk RNA sequencing, which captures averaged gene expression across mixed populations, single-cell analyses offer finer granularity and can resolve intra-tumoral heterogeneity, although at the cost of higher technical complexity and resource demands. A combination of both strategies may provide a more comprehensive understanding of the GABAergic immunoregulatory landscape. Additionally, the cellular sources of GABA in the tumor microenvironment remain poorly characterized; B cells, tumor cells, and even neurons may all contribute to its synthesis and secretion, forming a complex immunoregulatory network. In terms of therapeutic strategies, targeting GABA-synthesizing enzymes or receptors to enhance anti-tumor immunity has shown promise in animal models. However, most related studies remain at the preclinical stage, lacking systematic safety evaluations and clinical evidenc. Future research should systematically compare the immunoregulatory effects of GABA signaling across multiple tumor models to identify suitable therapeutic targets and responsive patient populations. Integrating advanced techniques such as single-cell genomics and spatial transcriptomics will be essential for elucidating the cellular sources and functional pathways of intratumoral GABA signaling. Furthermore, exploring synergistic mechanisms with existing immunotherapies—such as PD-1/PD-L1 inhibitors—may provide both theoretical support and practical strategies for the clinical translation of GABA-targeted interventions. Given the critical role of GABA in the nervous system, therapies targeting GABA signaling may cause various neurological side effects such as sedation, cognitive impairment, and motor dysfunction. These adverse effects mainly result from systemic modulation of GABA receptors in the central nervous system. To overcome this challenge, recent studies have explored strategies for tumor microenvironment-specific modulation (83). These include utilizing nanoparticle-based drug delivery systems for targeted transport, designing prodrugs that are locally activated within tumors to reduce systemic exposure, and developing highly selective agents targeting specific GABA receptor subtypes to minimize central nervous system involvement (84). Such approaches not only improve the safety and efficacy of treatment but also offer new avenues for precise immunotherapy targeting GABA signaling in tumors (85). Finally, future studies should also focus on how GABA-mediated intercellular communication dynamically regulates the immune landscape—balancing immune activation, suppression, and cell fate decisions—and how multi-target strategies combining GABA receptor modulators with other checkpoint inhibitors or metabolic regulators may help overcome tumor immune evasion. By leveraging the convergence of immunology, metabolism, and spatial genomics, the next phase of GABA-based immunotherapy research will provide new theoretical foundations and translational strategies for personalized cancer treatment.
